# Management of penicillin allergy in primary care: a qualitative study with patients and primary care physicians

**DOI:** 10.1186/s12875-021-01465-1

**Published:** 2021-06-11

**Authors:** Marta Wanat, Sibyl Anthierens, Christopher C. Butler, Louise Savic, Sinisa Savic, Sue H. Pavitt, Jonathan A. T. Sandoe, Sarah Tonkin-Crine

**Affiliations:** 1 Nuffield Department of Primary Care Health Sciences, University of Oxford, Radcliffe Observatory Quarter, Woodstock Road, Oxford, UK; 2 Department of Family Medicine and Population Health, University of Antwerp, Antwerp, Belgium; 3 Department of Anaesthesia, Leeds Teaching Hospitals NHS Trust, Leeds, UK; 4 Department of Clinical Immunology and Allergy, Leeds Teaching Hospitals NHS Trust, Leeds, UK; 5 Dental Translational and Clinical Research Unit, Faculty of Medicine and Health, University of Leeds, Worsley Building, Clarendon Way, Leeds, UK; 6 Healthcare Associated Infection Group, University of Leeds and Leeds Teaching Hospitals NHS Trust, Leeds, UK; 7 NIHR Health Protection Research Unit in Healthcare Associated Infections and Antimicrobial Resistance, University of Oxford, Oxford, UK

**Keywords:** Penicillin allergy, Qualitative, Primary care, Antibiotics

## Abstract

**Background:**

Six percent of patients are allergic to penicillin according to their medical records. While this designation protects a small number of truly allergic patients from serious reactions, those who are incorrectly labelled may be denied access to recommended first line treatment for many infections. Removal of incorrect penicillin allergy may have positive health consequences for the individual and the general population.

We aimed to explore primary care physicians’ (PCPs) and patients’ views and understanding of penicillin allergy with a focus on clinical management of infections in the face of a penicillin allergy record.

**Methods:**

We conducted an interview study with 31 patients with a penicillin allergy record, and 19 PCPs in the North of England. Data were analysed thematically.

**Results:**

Patients made sense of their allergy status by considering the timing and severity of symptoms. Diagnosis of penicillin allergy was reported to be ‘imperfect’ with PCPs relying on patient reports and incomplete medical records. PCPs and patients often suspected that an allergy record was incorrect, but PCPs were reluctant to change records. PCPs had limited knowledge of allergy services. PCPs often prescribed alternative antibiotics which were easy to identify. Both patients and PCPs differed in the extent to which they were aware of the negative consequences of incorrect penicillin allergy records, their relevance and importance to their lives, and management of penicillin allergy.

**Conclusions:**

PCPs and patients appear insufficiently aware of potential harms associated with incorrect penicillin allergy records. Some of the problems experienced by PCPs could be reduced by ensuring the details of newly diagnosed reactions to antibiotics are clearly documented. In order for PCPs to overturn more incorrect penicillin records through appropriate use of allergy services, more information and training about these services will be needed.

**Supplementary Information:**

The online version contains supplementary material available at 10.1186/s12875-021-01465-1.

## Background

Six percent of patients registered in general practice in England and 10% of patients in the US have a penicillin allergy label in their electronic health record [1, 2]. For patients who are truly allergic it is important that these records are correct, as this safeguards against serious/deadly consequences of consuming penicillin. Penicillins have been the most common cause of drug-inducted fatal and nonfatal anaphylaxis in the US and UK, but fatalities are still rare [3, 4]. However, it is also estimated that fewer than 10% of people who have a record of penicillin allergy are likely to be truly allergic to penicillins [5] and an increase in mortality has also been associated with having a penicillin allergy record [1].

Clinical confirmation of a historical diagnosis of penicillin allergy can be difficult for a number of reasons; there is often incomplete or inconsistent documentation of the reaction in medical records and patients may have no or limited recollection of the index event [6, 7]. The reasons for incorrect penicillin allergy labelling are complex. Sometimes symptoms of infection, such a rash caused by a viral illness, can be confused with an allergic reaction. Similarly, side effects to a penicillin such as nausea or diarrhoea can also lead to patients being labelled as allergic [8]. Understandably, PCPs have also concerns about missing penicillin allergy and causing serious reactions, especially if they have access to alternative antibiotics [9].

Penicillins are generally highly effective, narrow-spectrum, inexpensive antibiotics and are the first line recommended treatment for many infections. Patients with a penicillin allergy record are prescribed penicillin much less frequently and they received a range of different antibiotics instead [2]. In observational studies, patients with incorrect penicillin allergy records have been found to have longer hospital stays [10], increased risk of surgical site infections [11], treatment with potentially less effective and more costly antibiotics [2, 12], and increased rates of infection with Methicillin-resistant *Staphylococcus aureus* and *Clostridium difficile* compared to non-penicillin allergic patients [11–14]

These consequences have been recognised at policy level with the National Institute for Health and Care Excellence (NICE) advising clinicians to “double check patients with penicillin allergy” [15]. American Academy of Allergy, Asthma, and Immunology also highlighted that penicillin allergy is a top priority public concern [16]. Increasing numbers of studies are reporting on efforts to de-label patients with a penicillin allergy record in both primary and secondary care [17–19]. There is some understanding of patient and clinician views of penicillin allergy testing and “de-labelling” (removal of records that are found to be incorrect). Previous studies highlighted that PCPs felt that patients may not want to get tested [20] or may be worried about the safety of the test [20], while others described difficulties such as lack of time, not knowing what the referral criteria were [21] or lacking of access to any testing services [22]. Limited questionnaire-based studies highlighted that patients were not really aware of penicillin allergy testing services but were interested in testing [20, 23]. Our previous qualitative study explored in detail barriers and facilitators to patients attending, and PCPs referring for, penicillin allergy testing and subsequent use of penicillins [24]. This study highlighted a number of issues needing to be considered to enhance the effectiveness of de-labelling programmes [24], including the finding that clinicians felt that allergy testing could be beneficial but had limited experience of referring patients and similarly, that only patients who had experienced negative consequences of having penicillin allergy were motivated to get tested [24]. However, there is still limited understanding of both the patient and PCP perspectives on penicillin allergy and delabelling [25]. The current study builds on this work, by addressing an important research gap, identified by a number of recent reviews [8, 26] which higlighted that the views of patients and clinicians have been missing so far and called for qualitative studies to explore these views and help support de-labelling efforts [8, 26]. Thus, in the current study we have addressed the following research question: What are PCPs and patients views of penicilin allergy and their experiences of managing penicillin allergy in primary care?

## Methods

A qualitative study using semi-structured interviews in general practice in England. Methods have been previously described elsewhere but are summarised below [24].

### Recruitment

#### Patients

We used purposive sampling to identify participants with and without experience of penicillin allergy testing. Patients were identified using two methods: i) an audit of clinical records of patients with a (previous) record of penicillin allergy who attended a hospital drug allergy clinic for penicillin-allergy testing in the North of England between April 2015 and April 2017; ii) patients with a current record of penicillin allergy registered with general practices in the geographical area served by the allergy clinic.

#### Primary care physicians (PCPs)

We also sought to identify PCPs with and without experience of referring for penicillin allergy testing. PCPs were identified using three methods. Firstly, PCPs working in practices with patients who had undergone penicillin allergy testing in the hospital allergy clinic were identified and invited; secondly, physicians working in general practices in the geographical areas served by the hospital drug allergy clinic were invited; thirdly, PCPs who contacted the local microbiology service with relevant clinical queries during the study period were invited.

All potential participants were sent a recruitment pack and asked to contact the research team if they were interested in participating. After explaining the aims of the study, the interviewer answered any questions the participants had. If they were still willing to take part in the study, an interview was arranged. All participants gave written consent to take part in the study.

### Data collection

Two topic guides (one for patients and one for PCPs; provided in the supplementary materials) were developed based on the primary research questions and informed by the existing literature on penicillin allergy [25]. Throughout semi-structured telephone interviews, participants were encouraged to reflect on issues most important to them in relation to penicillin allergy and its impact [27]. The interviewer (MW) explained to participants that she was a non-clinical social science researcher carrying out research to improve health services and that she had no links with the allergy clinic. After obtaining consent, interviews were conducted, audio-recorded and transcribed verbatim. No repeat interviews were carried out.

### Analysis

Data collection and analysis took place concurrently. All interview data were analysed using inductive thematic analysis [28, 29]. One author, an experienced, female, post-doctoral qualitative researcher (MW), familiarised herself with all interviews, and her field notes and then coded initial transcripts line-by-line. Codes were compared with one another to create themes using constant comparison method [30]. This enabled a development of a draft coding framework (based on 19 interviews with patients and 9 with PCPs). The remaining interviews were then analysed using this flexible framework with changes made if needed. The data were managed in NVivo. In order to enhance credibility and trustworthiness of analysis, one third of transcripts was independently coded by STC [31]. The final codebook was agreed by all authors who included psychologists, a sociologist, a general practitioner, professors in health care research and hospital consultants with expertise in allergy, infection and delabelling. The participants were sent a summary of the findings. Interviews continued until data indicated thematic saturation in each participant group [32, 33]. This meant that no new findings were identified during the data collection stage; this was also assessed at the stage of analysis and meant that no new codes and themes were identified.

## Results

Approximately 100 patients were invited through one drug allergy clinic serving a large geographical area and 18 responded. In addition, over 100 patients were invited in primary care with 29 patients responding. This resulted in a total sample of 31 patients (sixteen patients were not interviewed as they could either not be contacted or contacted the team after the study reached data saturation).

Approximately 140 PCPs were invited via an audit of a drug allergy clinic, GP practices in the geographical areas served by the hospital drug allergy clinic and the local microbiology service. Nineteen participants responded (15 through the hospital microbiology service; 3 from primary care and 1 through the allergy clinic). This resulted in a total sample of 19 PCP participants.

A total of 50 interviews were conducted between December 2017 and August 2018 and lasted 20–60 min (average 46 min). The mean age of patients was 56 years (range 19–72) and 42 for PCPs (34–60). 80% of patients (n = 25) and 84% of PCPs (n = 16) were women.

A total of 11 themes were identified from the data. Of these, six themes concerned patient and PCP perspectives on penicillin allergy testing and subsequent use of penicillin containing antibiotics and are reported in our previous paper [24]. The remaining 5 themes focused on patient and PCP understanding of penicillin allergy and their experiences of managing penicillin allergy in primary care and are reported with supporting quotes below.

### Patients

#### Making sense of allergy

Patients varied in their recall of what had happened when they were initially diagnosed with a penicillin allergy, mainly due to time since diagnosis. Patients reported reflecting on the extent to which they believed their allergy record, taking into consideration the timing of symptoms and symptom severity. Patients who had developed symptoms immediately after taking penicillin and those who had symptoms perceived as severe, for example difficulty breathing, seemed to be particularly convinced that they were allergic. In contrast, symptoms perceived as mild, for example localised rash, appeared to make patients doubt their allergies, especially if the diagnoses was made a long time ago.

*I wasn’t ill. I don’t remember being really ill with it or anything like that, this rash, it just came and went. It wasn’t like I was desperately ill and rushed to hospital or anything, but I’ve never actually had the nerve to say I wasn’t allergic to penicillin in case I did have a reaction but, as I say, it was a long time ago and medical knowledge was very different in 1959 to what it is now (P29, Female, 72, current allergy record).*

Patients also queried why they had developed an allergy, especially if they were previously able to take penicillin. This was often described as a reaction “out of the blue”. Most patients were unsure whether their allergy status could change over time. Sometimes patients tried to make sense of their allergy by finding a trigger event which might have caused their allergy, such as having a heart transplant, or developing other allergies. Some questioned whether their allergy was hereditary and mentioned other family members having penicillin allergy; this in turn seemed to contribute to them accepting their diagnosis of penicillin allergy.

Well because my mum is allergic to it, I just thought it was genetic (P30, Female, 19, previous allergy record).

Finally, patients often had limited knowledge about the difference between an allergic reaction and a side effect. Most talked about their symptoms using these terms interchangeably. They seemed to have created a “working” definition of allergy, which meant that they have tried to make sense of what allergy is based on the symptoms they have experienced:

*I’ve always been aware of the sickness. I’ve always been aware of the skin reaction but it’s only that I’ve got older that I’ve been aware of the migraine style reaction [as part of the allergic reaction] (P6, Female, 44, current allergy record).*

#### Impact of allergy on managing health

Patients described to what extent penicillin allergy affected their lives. Patients, who believed that their allergy was a true allergy, unsurprisingly seemed to be more affected by their allergy status. They reported looking out for penicillin in prescribed medication, and reminding health care professionals that they were allergic each time they got a prescription.

*If I see anybody now I have to, although it’s on my medical records, I have to say I can’t take penicillin. I’ve got that in my phone and in my diary. If I had an accident, it’s down that I can’t take penicillin (P18, Female, 68, current allergy record).*

Similarly, participants who had chronic conditions or who were at risk of recurring infections described the impact of penicillin allergy. This included having a limited choice of antibiotics or worrying about running out of antibiotics in the future.

*It’s just not having those antibiotics available to me, it does concern me sometimes that’s what I’m concerned about is am I going to get an antibiotic that’s going to be able to get rid of it I guess (P16, Female, 47, current allergy record).*

In contrast, other participants described little or no effect of penicillin allergy on their life, especially if they rarely took antibiotics. Even participants who believed that penicillin was a useful antibiotic were unconcerned about not having access to penicillins. This seemed to be explained by the belief that alternative antibiotics were available to them, if needed. As a result, they did not think it was important to find out whether their allergy was a true allergy or not.

I don’t think I was concerned [about the diagnosis] because there are other antibiotics available (P20, Female, 58, current allergy record).

#### PCP influence on patients’ perceptions of allergy

Participants described how they were guided by their PCP in managing their allergy. This process often started when they were initially diagnosed, when patients accepted the diagnosis of penicillin allergy without question.

*I was only 13 or 14 and I took it face value. I went to see him, had the rash. He looked at me, looked at my notes, ‘You’ve had this penicillin injection. That’s what it is. It’s caused a reaction.’ I never questioned it as a teenage girl. I just thought he knows what he’s talking about, he said, ‘So you are allergic* to penicillin,’ and I just accepted that (P29, Female, 72, current allergy record).

As a result, the majority of patients reported that they never really discussed their allergy with their PCP and that alternative antibiotics were always prescribed. They appeared to accept this, seeing the PCP as an expert in choosing the right treatment.

*It was just kind of, ‘Are you allergic to anything? Oh wait, you’re allergic to penicillin.’ Then they just wouldn’t prescribe it. That was about it, they never really discussed taking it further with investigations or anything (P3, Female, 47, previous allergy record).*

In addition, some patients reported that they would take their PCP advice in relation to whether testing or in fact taking penicillin, despite current allergy record, would be beneficial for them.

*I suppose I would have to rely on what the expert told me. If they said I needed it to save my life, then I would take it but if they could offer me an alternative I think I would probably rather explore the alternatives given that I know that I could come out with a small reaction to the penicillin (P30, Male, 36, current allergy record).*

### PCPs

#### Uncertainties around diagnosing penicillin allergy

PCPs described ways of identifying patients with allergies. They routinely checked patients’ allergies on their medical records and described how the electronic system reminded them about patient allergies at the point of issuing a prescription. They perceived this as an important safety measure. In addition, some PCPs routinely asked patients about their allergies and recorded them if patients reported them. While some PCPs relied mainly on the system, others felt that checking with the patient each time was essential.

*Well, I always ask, because it makes me feel better. There is actually a system where if you prescribe an antibiotic and they’re allergic to it, it does flash up and says, ‘There is a registered intolerance.’ But I’m just a bit anxious that I might miss that. There are so many pop ups that sometimes you might just become a little bit desensitised to them. I don’t want to kill anyone, I’m terrified that if I give somebody an antibiotic and they take it and they’re allergic, that they are going to have an anaphylactic reaction and they’re gonna die (P8, 2 years of experience).*

PCPs tried to gather information from patients about the details of their reactions both new and previously reported; the aim of this was to check whether it was a true allergy or just a side effect but most commonly to understand what happened and to record this information accurately and comprehensively in patient’s medical records.

*You have to record what sort of allergic reaction the patient had, you complete it so that the next person looking at it will have a clear idea of what was the allergic reaction or was it a side effect or not (P3, 12 years of experience).*

However, PCPs also reported that the electronic records system was imperfect as it often did not differentiate between a side effect and an allergy, which caused problems later on.

*It is difficult on our system to distinguish between allergies and side effects because it just comes up as adverse reactions so they would still not get it because it is coming up as an adverse reaction (P12, 20 years of experience).*

As a result, PCPs often doubted whether the allergy status was correct. This was especially the case when the information from patients and their records was very brief and based on a historical diagnosis (e.g. records just stating “allergic” or patients not being able to recall any details about the allergic reaction) or when the medical record was based on family history rather than personal experience of an allergic reaction. Many were aware that penicillin allergy is over diagnosed.

*You can't be sure and that's the problem isn't it? You'd like to be able to say 'Ok this patient is definitely penicillin allergic' but if the patient has a vague recollection that around the same time that they had penicillin they had a rash and there's nothing documented in the notes, or if there is something documented in the notes it's not consistent with what the patient is saying it's very difficult to put that in – make a diagnosis (P4, 28 years of experience).*

PCPs often commented on what they thought constituted an allergic reaction. While there was consensus about an anaphylactic reaction constituting a true allergy, beyond that PCPs differed in what symptoms they considered to be an allergic reaction and included symptoms such as feeling unwell or patients having gastrointestinal symptoms. In addition, PCPs described how some symptoms, such as rash, made it very difficult to diagnose allergy.

*Obviously, there are symptoms of allergy like anaphylaxis, the difficulty is those slightly vague ones where they’ve got a bit of a rash, so actually is that a bit of an urticarial rash that you’ve got? Is it a bit of a non-specific viral rash in a child? It’s difficult to say (P6, 6 years of experience).*

#### Making prescribing decisions for patients with penicillin allergy

PCPs commented on the ease of finding and giving an alternative antibiotic for patients with penicillin allergy. This was often facilitated by ease of using guidelines, or being able to ask for advice.

*Prescribing] won’t be that complicated. There’s always option B. As I said, it’s actually from our local guidance that gave us the options. It’s all very well established. It’s not complicated to find an option (P5, 19 years of experience).*

Finding an alternative antibiotic was seen as difficult for only a minority of patients; mainly patients who either had recurrent infections or a record of multiple allergies.

The worry is [having] more than one allergy to an antibiotic; that becomes a problem (P5, 19 years of experience).

Despite doubting patient’s allergies, PCPs often made the decision not to prescribe penicillin as they felt that that they could never be 100% sure that it was not a true allergy. They described their worry about “making a mistake” and causing someone to have anaphylactic shock and as a result facing legal action. They also felt that if something was recorded on patient’s medical records, they could not “ignore” it.

*Obviously first of all do no harm and I don’t want the patients to come to harm, of course I don’t but then similarly I think it’s a very brave/foolish maybe PCP that chooses to ignore a documented penicillin allergy and to give penicillin […] and so that just makes me feel nervous because if you get it wrong and it causes a death, that sits very uncomfortably as I’m sure it would when I was standing in front of the GMC and they kind of said well why on earth when it clearly says there’s a penicillin allergy on this patient’s records, did you give penicillin? It’s kind of indefensible isn’t it really (P11, 12 years of experience).*

In contrast, minority of PCPs felt confident to challenge allergy label and prescribe penicillin for patients with a record of penicillin allergy; this was either for patients for whom they struggled to prescribe or for patients for whom they believed that penicillin was the best antibiotic. This was sometimes triggered by advice from microbiology or their experience of successfully challenging allergy record previously.

*If a penicillin-based antibiotic is the best option, I sometimes have a conversation with the patients about whether they are happy to have a trial or not and the vast majority that I have done that to have taken the stuff and have been absolutely fine. You do it on a case-by-case basis depending on what you think is the most appropriate antibiotic for that patient and how you think that person will handle the situation if they start to show allergic symptoms again or not (P7, 26 years of experience).*

A minority of PCPs also had experience of challenging allergy records by referring patients for formal testing in secondary care. Most PCPs were often uncertain about referral criteria and what the testing involved.

I don’t know in detail the services available and I suppose it’s just not knowing what is appropriate for them to see (P1, 2 years of experience).

PCPs were aware of the consequences of incorrect allergy labels, including not providing patients with optimal treatment, reducing number of antibiotic options for patients in the future, and potentially prescribing more expensive antibiotics or agents with more side effects. However, they rarely described any negative consequences relevant to patients outside of managing them in primary care (e.g. longer hospital stays). Also, the difficulties which PCPs did report, were not perceived by them as present in their day-to-day management of patients. In turn, they felt they had limited importance when making prescribing decisions in the present. Only a minority mentioned antibiotic resistance as an important consideration.

*I definitely think it is an issue, it is a real issue, as I am very aware that we are over-labelling patients with penicillin allergy that do not have an allergy at all and I am aware of the fact that studies are showing that patients will fare worse, if they are labelled as having penicillin allergy […] so I think it is a really big issue but on a day-to-day level we get round it at the moment without too much difficulty (P12, 20 years of experience)*

## Discussion

This study fills an important research gap by providing an in-depth understanding of patient and PCPs views of penicillin allergy and its management in primary care. It compliments quantitative descriptive work, which has identified the scale of the problem, by bringing to the fore the perspectives of the people that really matter, namely the prescribers and the patients themselves. Their accounts are critical to achieving a deeper understanding of the barriers and opportunities for improved clinical practice for patient benefit.

This study highlights a number of novel findings in relation to patients’ views of penicillin allergy. Patients tried to make sense of their allergy status by considering timing and severity of symptoms. Overall, they had limited understanding of penicillin allergy, including what constituted symptoms of allergy versus side effect. It seemed that they tried to create their own working definition of allergy based on their direct experience. These issues have not been explored before, with only one previous study briefly assessing patients’ knowledge of penicillin allergy and highlighting that patients were not aware that drug allergy can wane over time [23]. Our study highlights a need for better awareness of the importance of incorrect penicillin allergy records by both patients and PCPs. PCPs discussions with patients about their penicillin allergy might be beneficial, with a particular focus on the difference between a side effect and allergic reactions. This knowledge might also increase patient and PCP motivation to engage with the process of delabelling.

Both patients and PCPs differed in the extent to which they were aware of negative consequences of incorrect penicillin allergy records, their relevance and in turn importance to their lives and management of penicillin allergy. Not surprisingly, patients and PCPs who experienced negative consequences of penicillin allergy were in turn more aware of them and saw them as more relevant. However, some patients were not aware of the negative consequences and some PCPs did not see them as important or relevant in primary care. Both patients and PCPs may also benefit from more information about potential negative consequences of penicillin allergy. While this does not mean that this will and should lead to delabelling decision, it may help PCPs and patients to have more informed discussions about managing penicillin allergy in primary care.

In line with previous research on PCPs experiences of managing drug allergy in general [34], in the current study, PCPs described a number of difficulties in diagnosing penicillin allergy. They highlighted that medical records and patient reports of events were often incomplete, which contributed to diagnostic uncertainty. This meant that while PCPs doubted that some patients were truly allergic, they were also reluctant to change medical records. The current study highlighted two issues, which seemed to influence PCP decision making in relation to penicillin prescribing. Firstly, PCPs found it easy to manage the majority of patients with a penicillin allergy as guidelines were clear on what to prescribe as second-line and the alternatives were easily available. Secondly, this decision was often reinforced by PCPs being worried about inadvertently causing an allergic reaction. These issues have been raised by previous research conducted in secondary care; one study reported that while almost 60% of hospital consultants and doctors in training agreed that it is always better to “play it safe” and not use beta-lactams in patients labelled penicillin-allergic [9], more than 90% also disagreed with the statement that “accurate allergy history is not important when making prescribing decisions because there are many alternatives to beta-lactams that have comparable efficacy and safety” [9]. Similarly, another study also found that one of the major factors influencing prescribing behaviour was the ready availability of alternate antibiotics [35]. The current study extends this knowledge and highlights factors specific to primary care.

Our study also highlights the need to support PCPs in order for them to be able to carry out delabelling. Recently, new models of delabelling have been proposed; for example, through supporting clinicians to use of an algorithm, a structured interview or a clinical decision tool for patients reporting drug allergy to establish whether harm was caused by medication [36–38]. Using a decision support tool or algorithm might be useful for PCPs in order to ensure a consistent approach across primary care. This might be a particularly beneficial approach for low risk patients, for example patients with likely non-immunological reactions who still hold a record of penicillin allergy [29–31]. For patients when there is still uncertainty, this approach to de-labelling could be complemented by secondary care specialist allergy services. A number of barriers need to be addressed though. First, there is a need to ensure that PCPs have access to penicillin allergy clinics. Secondly, where these are available, PCPs need to be aware of such services and know the referral process and criteria. Thirdly and perhaps most importantly, PCPs need to be convinced in the benefits of allergy services for their patients [25].

### Strengths and limitations

This is the first qualitative interview study to provide an in-depth understanding of patient and PCP views and experiences of managing penicillin allergy in UK primary care. It builds on our previous work which focused on identifying barriers and facilitators to penicillin allergy testing and subsequent antibiotic use [24]. As previous studies used mainly survey designs and only included hospital physicians, this study fills an important gap by providing a comprehensive account of patient and PCP perspectives. The study also benefited from a multidisciplinary team working on the analysis which enhanced the trustworthiness of data. However, both patients and PCP were a convenience sample recruited from one region in England which means that these findings may have to be interpreted cautiously in terms of their transferability to other settings.

## Conclusions

Both patients and PCPs need to be provided with information about the negative consequences of incorrect penicillin allergy records, their relevance and supported to facilitate processes of delabelling, where appropriate.

## Supplementary Information

**Additional file 1: Appendix** PCP Interview Guide.

## Data Availability

The datasets generated and/or analysed during the current study are not publicly available due to confidentiality reasons but are available from the corresponding author on reasonable request.

## Abbreviations

PCP: Primary care physician
NICE: National Institute for Health and Care Excellence
