# Supplementary material for: Management of penicillin allergy in primary care: a qualitative study with patients and primary care physicians
Source: BMC Fam Pract. 2021 Jun 11;22:112. doi: 10.1186/s12875-021-01465-1 (PMC8194168; doi:10.1186/s12875-021-01465-1)
Supplement: Supplementary file 1 — Additional file 1: Appendix PCP Interview Guide. [file 12875_2021_1465_MOESM1_ESM.docx]

**Appendix**

**PCP Interview Guide**

1. Can you tell me about how you usually identify patients who have a penicillin allergy?

Prompts: How is penicillin allergy recorded in patient notes? How easy it is to obtain this information? How often do patients report a penicillin allergy themselves?

2. Can you tell me about your experience of caring for patients who have a record of penicillin allergy?

Prompts: How frequently do you see patients with an allergy to penicillin? How easy or difficult do you feel it is to make prescribing decisions for these patients? How much do you know about penicillin allergy?

3. Can you tell me what types of infections patients commonly present with for which you would prescribe antibiotics?

Prompts: For what other conditions would you consider prescribing antibiotics for these patients?

4. How frequently do you check a patient’s allergy record before prescribing antibiotics?

Prompts: How do you check this? By asking the patient and/or checking their notes? What happens if a patient reported allergy does not match their record? Or vice versa? How important do you think it is to check for an allergy record?

5. What sources of information do you refer to when deciding what antibiotic to prescribe to these patients?/How do you make decision about which antibiotic to prescribe to these patients?

Prompts: Can you tell me about any guidelines you are aware of for making prescribing decisions for persons with penicillin allergy? How confident do you feel about prescribing for these patients?

6. What sort of discussions do you have with patients about penicillin allergy?/How do you talk to patients about penicillin allergy?

Prompts: How do you explain allergy to your patient? How do you discuss the type of antibiotic choice with patients? How confident are you in having these discussions?

7. What sorts of word do you use when you talk about allergy? (for example sensitivity/hypersensitivity, side effect).

Prompts: How would you explain a penicillin allergy to a patient? Would you consider these words to be equivalent?

8. Can you tell me about a time where you have identified a penicillin allergy and changed a patient’s medical record based on this?

Prompts: How have you previously identified a penicillin allergy? What would make you add an allergy to a patient’s medical record? How easy is it to change a patient’s penicillin allergy record? How confident are you in changing a patient’s medical record?

9. Can you tell me about a time where you have queried whether a patient is actually allergic to penicillin?

Prompts: In what sort of situation do you doubt that a patient has a genuine allergy to penicillin? What makes you query a penicillin allergy record? How has this influenced your prescribing decision?

10. What do you think are the consequences of patients having an incorrect penicillin allergy label?

Prompts: What disadvantages are there for patients? What disadvantages are there for your practice? Any other disadvantages?

11. What action do you take if you think a penicillin allergy record is incorrect?

Prompts: When would you choose to refer someone to an allergy service? Are there any barriers to changing a penicillin record?

**Patient Topic guide**

1. Can you tell me about when you were first diagnosed with an allergy to penicillin?

Prompts: How did you find out you had a penicillin allergy? Who told you that you were allergic to penicillin? What did you think about it?

2. Can you tell me about any previous reactions you have had to penicillin, or drugs containing penicillin in the past?

Prompts: How did your body react to penicillin? What sort of symptoms did you get? How long after taking penicillin did you get a reaction?

3. How do you manage your penicillin allergy?

Prompts: What sorts of things do you do to avoid taking penicillin? What do you do to make sure others know about your allergy? What advice have you received about managing your penicillin allergy?

4. How would you describe your penicillin allergy to a friend?

Prompts: How would you explain what a penicillin allergy is? How would you tell them about the medications you can and can’t take?

5. Can you tell me about any other allergies that you have?

Prompts: What would indicate to you that you were allergic to something? What sort of symptoms do you think you might experience?

5. In what situations do you talk about your penicillin allergy with your GP?

Prompts: How frequently do you or your GP talk about your allergy? When do you usually tell a doctor about your allergy? Does your GP know that you are allergic to penicillin?

6. Can you tell me about any other words that you or your doctor uses to describe your penicillin allergy?

Prompts: Do you or your doctor talk about sensitivity or hypersensitivity to penicillin? What do you think these terms mean?

7. Can you tell me some occasions when you have had to take antibiotics?

Prompts: What conditions was this for? Did you and your GP discuss your penicillin allergy? What types of antibiotic have you taken previously? Do you have any conditions where you need to take antibiotics regularly? Do you have any concern about taking antibiotics?

8. Is there anything else you would like to mention that we haven’t talked about?
